# DLX6-AS1 activated by H3K4me1 enhanced secondary cisplatin resistance of lung squamous cell carcinoma through modulating miR-181a-5p/miR-382-5p/CELF1 axis

**DOI:** 10.1038/s41598-021-99555-8

**Published:** 2021-10-25

**Authors:** Xu Zhao, Jizhao Wang, Rui Zhu, Jing Zhang, Yunfeng Zhang

**Affiliations:** 1 Department of Radiation Oncology, The First Affiliated Hospital of Xi’an Jiaotong University, Xi’an, 710061 Shaanxi China; 2 Department of Thoracic Surgery, The First Affiliated Hospital of Xi’an Jiaotong University, No.277, Yanta West Road, Xi’an, 710061 Shaanxi China

**Keywords:** Cancer, Lung cancer, Non-small-cell lung cancer

## Abstract

Cisplatin (CDDP) based chemotherapy is widely used as the first-line strategy in treating non-small cell lung cancer (NSCLC), especially lung squamous cell carcinoma (LUSC). However, secondary cisplatin resistance majorly undermines the cisplatin efficacy leading to a worse prognosis. In this respect, we have identified the role of the DLX6-AS1/miR-181a-5p/miR-382-5p/CELF1 axis in regulating cisplatin resistance of LUSC. qRT-PCR and Western blot analysis were applied to detect gene expression. Transwell assay was used to evaluate the migration and invasion ability of LUSC cells. CCK-8 assay was used to investigate the IC50 of LUSC cells. Flow cytometry was used to test cell apoptosis rate. RNA pull-down and Dual luciferase reporter gene assay were performed to evaluate the crosstalk. DLX6-AS1 was aberrantly high expressed in LUSC tissues and cell lines, and negatively correlated with miR-181a-5p and miR-382-5p expression. DLX6-AS1 expression was enhanced by H3K4me1 in cisplatin resistant LUSC cells. Besides, DLX6-AS1 knockdown led to impaired IC50 of cisplatin resistant LUSC cells. Furthermore, DLX6-AS1 interacted with miR-181a-5p and miR-382-5p to regulate CELF1 expression and thereby mediated the cisplatin sensitivity of cisplatin resistant LUSC cells. DLX6-AS1 induced by H3K4me1 played an important role in promoting secondary cisplatin resistance of LUSC through regulating the miR-181a-5p/miR-382-5p/CELF1 axis. Therefore, targeting DLX6-AS1 might be a novel way of reversing secondary cisplatin resistance in LUSC.

## Introduction

Lung cancer, divided into small cell lung cancer (SCLC) and non-small cell lung cancer (NSCLC), is one of the most common malignancies and the most common cause of cancer death in the world, with almost 1.8 million newly diagnosed cases every year^1^. Lung squamous cell carcinoma (LUSC), one of the major subtypes of NSCLC, accounts for nearly 30% of all confirmed cases of NSCLC, with the characteristics of rapid growth, quick metastasis and high mortality^2^. Although relatively sensitive to chemotherapy and radiotherapy, the 5-year survival rate for patients with LUSC is merely about 18%^3^. Platinum-based systemic chemotherapy is still the standard treatment for LUSC patients, but it often leads to clinical treatment failure due to the development of secondary chemoresistance^4^. Therefore, it is urgent to identify the novel molecular targets that affect the secondary chemoresistance of LUSC patients.

Long non-coding RNAs (lncRNAs) are non-protein-coding RNAs with a length of more than 200 nucleotides, accounting for more than 90% of all transcriptome^5^. At present, increasing evidence has shown that lncRNAs, which could act as competing endogenous RNA (ceRNA), play a crucial role in the occurrence and progression of malignant tumors via competitively binding to microRNAs (miRNAs) to regulate the expression of target mRNAs, and their aberrant expression are usually closely related to the proliferation, invasion, metastasis, and chemoresistance of tumor cells^6–8^. For instance, lncRNA LINC01963 increases TMEFF2 expression to inhibit the progression of pancreatic carcinoma cells via targeting miR-641^9^. LncRNA TALC promotes the expression of O6-methylguanine DNA methyltransferase (MGMT) by competitively binding miR-20b to activate the c-MET/STAT3/p300 axis, which leads to temozolomide (TMZ) resistance in glioblastoma^10^. The lncRNA DLX6-AS1, located at chromosome 7q21.3, is a 1990 bp non-coding transcript^11^. Recently, growing reports have indicated that DLX6-AS1 could interact with the target miRNAs to regulate the progression and treatment resistance of multiple cancers, including lung adenocarcinoma (LUAC)^12^, ovarian cancer^13^, hepatocellular cancer (HCC)^14^, glioma^15^ and bladder cancer^16^. Nevertheless, the potential role of DLX6-AS1 in LUSC chemoresistance has rarely been entirely investigated.

miRNAs represent a class of short non-coding RNA molecules, with a length of 19 to 25 nucleotides, which negatively regulate gene expression at both post-transcriptional and translational levels via interacting with the 3′-untranslated region (3′-UTR) of target messenger RNAs (mRNAs)^17^. Recent evidence indicated that miRNAs played a crucial role in tumorigenesis, metastasis, and chemoresistance of multiple cancers^18,19^. miR-181a-5p was confirmed to act as a tumor suppressor in many cancers, including breast cancer^20^, NSCLC^21^, and colorectal cancer^22^. In addition, the aberrant expression of miR-382-5p was reported to be closely related to proliferation, invasion, and migration of many types of cancers^23–25^. Nevertheless, the specific function of miR-181a-5p and miR-382-5p in chemoresistance of LUSC has not been clearly elucidated.

CUGBP, Elav-like family member 1 (CELF1) is an RNA binding protein regulating pre-mRNA alternative splicing, mRNA editing, and translation^26^. Recently, CELF1 was demonstrated to be abundantly expressed in various cancers, and its aberrant expression was closely correlated to growth, metastasis, as well as chemoresistance in multiple malignancies^27–29^. However, the involvement of CELF1 in the acquired cisplatin resistance of LUSC remains unclear.

Therefore, the main aim of this research was to explore the underlying function of lncRNA DLX6-AS1 in the progression of LUSC and to reveal the possible mechanisms of which DLX6-AS1 correlated with platinum-based secondary chemoresistance in LUSC. Furthermore, we also investigated in the present study the interaction of DLX6-AS1 with miR-181a-5p, miR-382-5p, and CELF1 in this process. Taken together, our findings suggested that DLX6-AS1 served as a new molecular target for modulating the secondary chemoresistance of LUSC, which might provide a promising therapeutic target for LUSC treatment.

## Results

### DLX6-AS1 was highly expressed in LUSC tissues and was negatively correlated with miR-181a-5p and miR-382-5p expression

**Figure 1:**
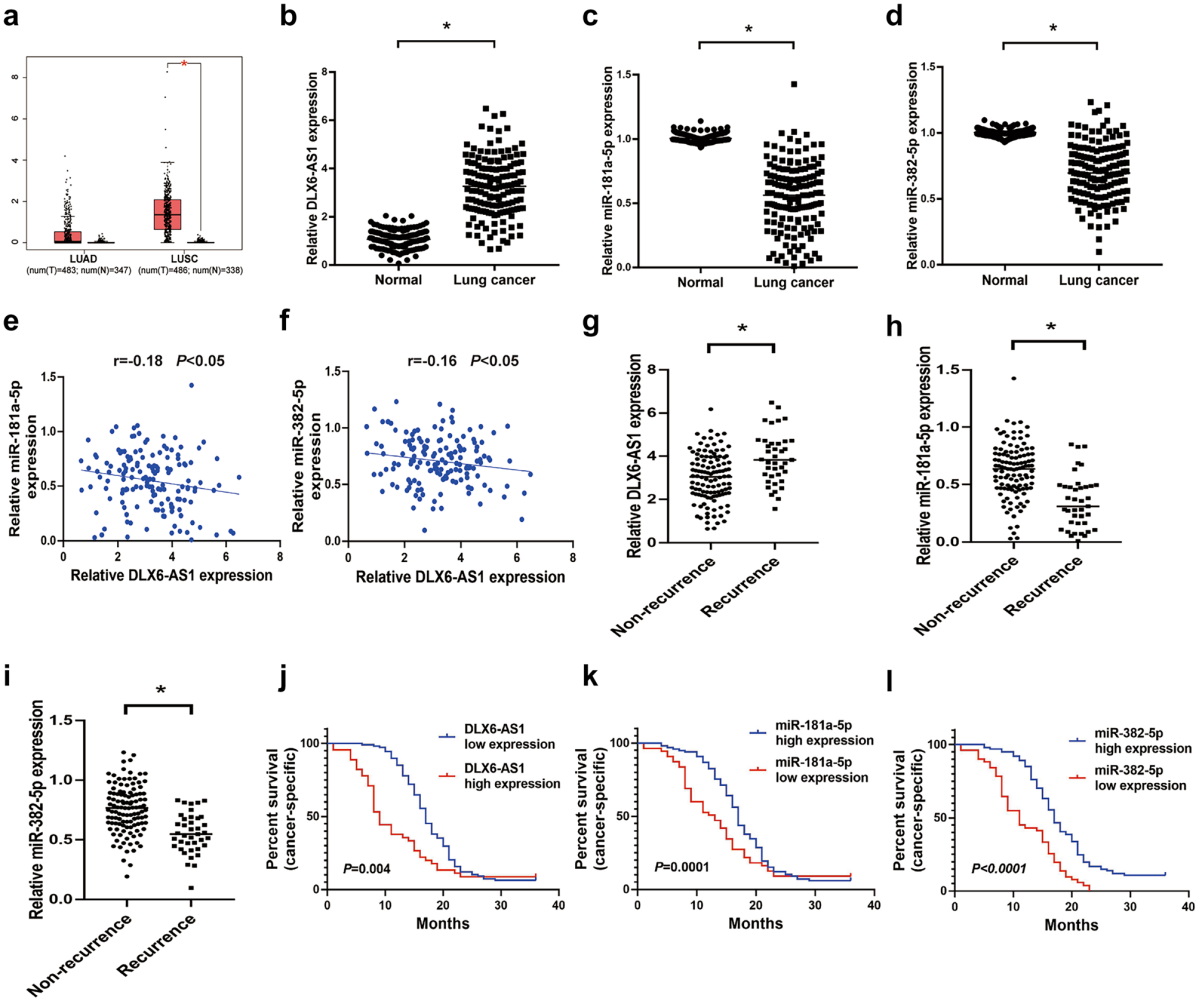
DLX6-AS1 was highly expressed in LUSC tissues and was negatively correlated with miR-181a-5p and miR-382-5p expression. (**a**) The relative expression of DLX6-AS1 in lung adenocarcinoma tissues (LUAD, n = 483) and normal tissues (n = 347), as well as in lung squamous cell carcinoma tissues (LUSC, n = 486) and normal tissues (n = 338) based on GEPIA. (**b**) qRT-PCR analysis for the relative expression of DLX6-AS1 in LUSC tissues (n = 153) and para-cancer tissues (n = 153). (**c**) qRT-PCR analysis of miR-181a-5p expression in LUSC tissues (n = 153) and para-cancer tissues (n = 153). (**d**) qRT-PCR analysis of miR-382-5p expression in LUSC tissues (n = 153) and para-cancer tissues (n = 153). (**e**) Spearman correlation analysis for the expression levels of DLX6-AS1 and miR-181a-5p in LUSC tissues. (**f**) Spearman correlation analysis for the expression levels of DLX6-AS1 and miR-382-5p in LUSC tissues. (**g**) qRT-PCR analysis for the relative expression of DLX6-AS1 in local recurrence LUSC tissues (n = 39) and non-recurrence LUSC tissues (n = 114). (**h**) qRT-PCR analysis of miR-181a-5p expression in local recurrence LUSC tissues and non-recurrence LUSC tissues. (**i**) qRT-PCR analysis of miR-382-5p expression in local recurrence LUSC tissues (n = 39) and non-recurrence LUSC tissues (n = 114). (**j**) The cancer-specific survival of LUSC patients was estimated by Kaplan–Meier survival analysis according to DLX6-AS1 expression levels. (**k**) The cancer-specific survival of LUSC patients was estimated by Kaplan–Meier survival analysis according to miR-181a-5p expression levels. (**l**) The cancer-specific survival of LUSC patients was estimated by Kaplan–Meier survival analysis according to miR-382-5p expression levels. *P < 0.05.

Based on Gene Expression Profiling Interactive Analysis (GEPIA), we noticed that DLX6-AS1 was significantly differentially expressed in LUSC but not LUAD (Fig. 1a). Then we validated DLX6-AS1 expression in vivo and found that it was highly expressed in LUSC tissues (153 cases) compared with para-cancer tissues (153 cases; Fig. 1b). Then DIANA LncBase Experimental v.2 prediction showed that miR-181a-5p and miR-382-5p were the potential downstream targets of DLX6-AS1. Further in vivo experiments showed that miR-181a-5p and miR-382-5p expression were decreased in LUSC tissues (153 cases; Fig. 1c,d). In addition, DLX6-AS1 expression was negatively correlated with miR-181a-5p and miR-382-5p expression (Fig. 1e, f). Furthermore, we found that DLX6-AS1 level was increased in local recurrence LUSC tissues (39 cases; Fig. 1g), while miR-181a-5p and miR-382-5p levels were apparently decreased (Fig. 1h,i). Moreover, survival analysis showed that DLX6-AS1 overexpression led to worse cancer-specific survival (Fig. 1j), whereas miR-181a-5p and miR-382-5p led to better cancer-specific survival (Fig. 1k,l).

### DLX6-AS1 knockdown inhibited proliferation, migration, invasion and promoted apoptosis of LUSC cells

**Figure 2:**
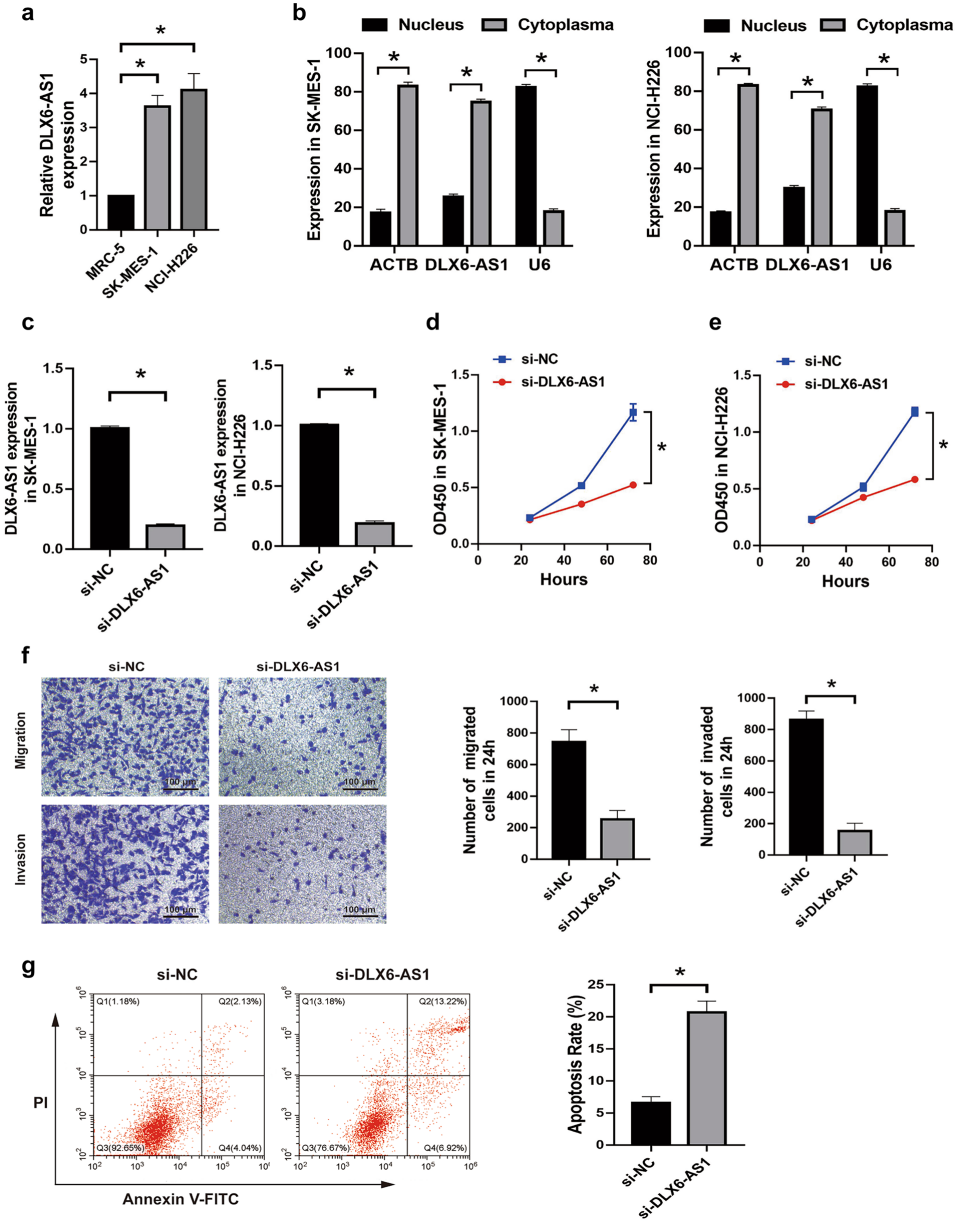
DLX6-AS1 knockdown inhibited proliferation, migration, invasion and promoted apoptosis of LUSC cells. (**a**) qRT-PCR analysis for the relative expression of DLX6-AS1 in SK-MES-1, NCI-H226, and MRC-5 cell lines. (**b**) qRT-PCR analysis for the relative expression of DLX6-AS1 in the cytoplasm or nucleus of SK-MES-1 and NCI-H226 cell lines. (**c**) The relative expression of DLX6-AS1 transfected with si-DLX6-AS1 or si-NC in SK-MES-1 and NCI-H226 cell lines was estimated by qRT-PCR analysis. (**d,e**) CCK-8 assay was performed to detect the proliferation of SK-MES-1 (**d**) and NCI-H226 (**e**) cells transfected with si-DLX6-AS1 or si-NC. (**f**) Transwell analysis of SK-MES-1 cells transfected with si-DLX6-AS1 or si-NC. (**g**) Apoptotic rate of SK-MES-1 cells transfected with si-DLX6-AS1 or si-NC. *P < 0.05.

Further in vitro experiments indicated that DLX6-AS1 overexpressed in SK-MES-1 and NCI-H226 cell lines than that in MRC-5 cell line (Fig. 2a), besides DLX6-AS1 mostly expressed in the cytoplasm of LUSC cells (Fig. 2b). Then we inhibited the expression of DLX6-AS1 in SK-MES-1 and NCI-H226 cells (Fig. 2c). CCK-8 results suggested that DLX6-AS1 knockdown led to decreased proliferation ability in SK-MES-1 and NCI-H226 cells (Fig. 2d,e). In addition, transwell assay showed that DLX6-AS1 knockdown significantly suppressed the migration and invasion of SK-MES-1 cells (Fig. 2f). Furthermore, apoptosis rate was obviously increased by attenuating DLX6-AS1 expression in SK-MES-1 cells (Fig. 2g). Thus, these results indicated that DLX6-AS1 served as an oncogene to promote the growth and progression of LUSC cells.

### DLX6-AS1 negatively regulated the expression of miR-181a-5p and miR-382-5p

**Figure 3:**
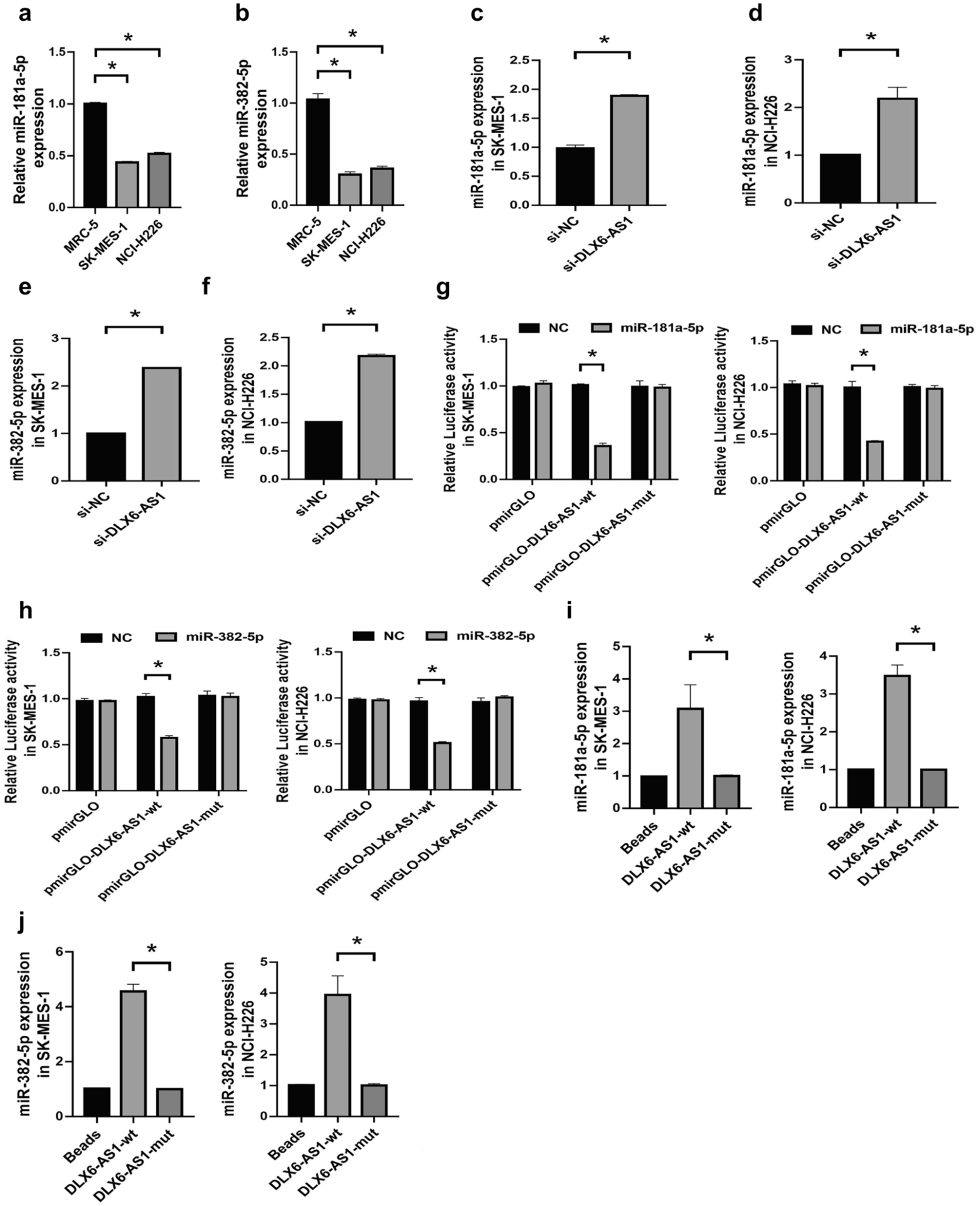
DLX6-AS1 negatively regulated the expression of miR-181a-5p and miR-382-5p. (**a,b**) qRT-PCR analysis for the relative expression of miR-181a-5p (**a**) or miR-382-5p (**b**) in SK-MES-1, NCI-H226, and MRC-5 cell lines. (**c,d**) The relative expression of miR-181a-5p in SK-MES-1 (**c**) and NCI-H226 (**d**) cell lines transfected with si-DLX6-AS1 or si-NC was estimated by qRT-PCR analysis. (**e,f**) The relative expression of miR-382-5p in SK-MES-1 (**e**) and NCI-H226 (**f**) cell lines transfected with si-DLX6-AS1 or si-NC was estimated by qRT-PCR analysis. (**g**) The binding relationship between DLX6-AS1 and miR-181a-5p in SK-MES-1 or NCI-H226 cells was verified by performing Dual luciferase reporter gene assay. (**h**) The binding relationship between DLX6-AS1 and miR-382-5p in SK-MES-1 or NCI-H226 cells was verified by performing Dual luciferase reporter gene assay. (**i**) The interaction between DLX6-AS1 and miR-181a-5p in SK-MES-1 or NCI-H226 cells was estimated via RNA pull down assay. (**j**) The interaction between DLX6-AS1 and miR-382-5p in SK-MES-1 or NCI-H226 cells was estimated via RNA pull down assay. *P < 0.05.

Then we detected the expression of miR-181a-5p and miR-382-5p in LUSC cells and found that miR-181a-5p and miR-382-5p expression was at lower levels in SK-MES-1 and NCI-H226 cell lines than that in MRC-5 cell line (Fig. 3a,b). Furthermore, DLX6-AS1 knockdown resulted in increased expression of miR-181a-5p and miR-382-5p (Fig. 3c–f), indicating that miR-181a-5p and miR-382-5p might be the target of DLX6-AS1. Then we performed Dual luciferase reporter gene assay and noticed that DLX6-AS1 could interact with miR-181a-5p and miR-382-5p in SK-MES-1 and NCI-H226 cells (Fig. 3g,h). Further RNA pull-down assay showed that DLX6-AS1 was directly binding with miR-181a-5p and miR-382-5p in LUSC cells (Fig. 3i,j). These findings suggested that DLX6-AS1 negatively regulated miR-181a-5p and miR-382-5p in LUSC cells.

### DLX6-AS1 was induced by H3K4me1 in cisplatin resistant LUSC cells

**Figure 4:**
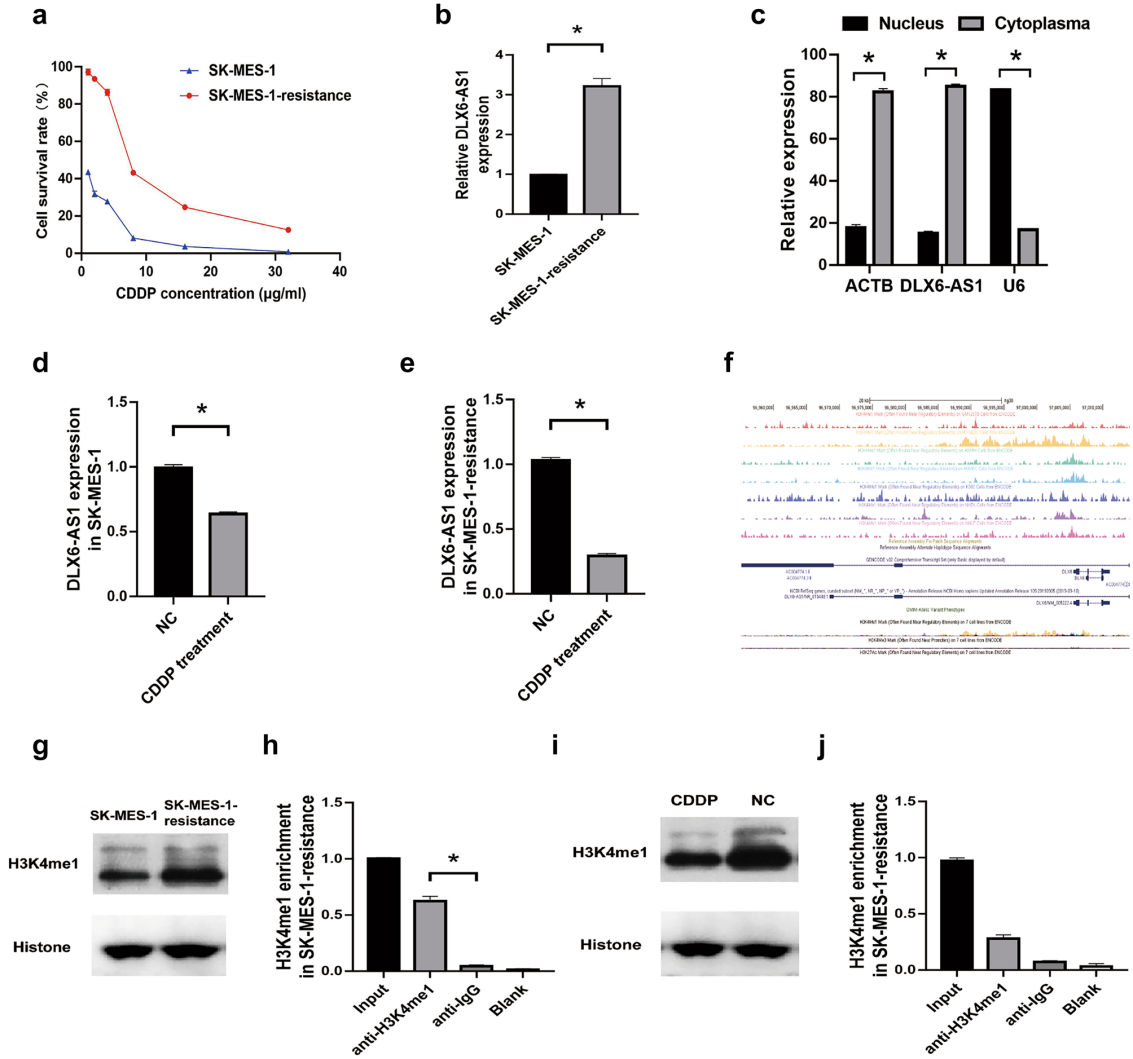
DLX6-AS1 was induced by H3K4me1 in cisplatin resistant LUSC cells. (**a**) The IC50 of CDDP in SK-MES-1 or SK-MES-1-resistance cell lines was detected by conducting CCK-8 assay. (**b**) qRT-PCR analysis for the relative expression of DLX6-AS1 in SK-MES-1 or SK-MES-1-resistance cell lines. (**c**) qRT-PCR analysis for the relative expression of DLX6-AS1 in the cytoplasm or nucleus of SK-MES-1-resistance cell line. (**d,e**) qRT-PCR analysis for the relative expression of DLX6-AS1 in SK-MES-1 (**d**) or SK-MES-1-resistance (**e**) cell lines treated with or without CDDP. (**f**) The binding relationship between the promoter region of DLX6-AS1 and H3K4me1 was predicated by UCSC. (**g**) The relative expression of H3K4me1 in SK-MES-1 or SK-MES-1-resistance cells was estimated by Western blot analysis. (**h**) ChIP assay analysis for the enrichment of H3K4me1 in SK-MES-1-resistance cells. (**i**) The relative expression of H3K4me1 in SK-MES-1-resistance cells treated with CDDP was estimated by Western blot analysis. (**j**) ChIP assay analysis for the enrichment of H3K4me1 in SK-MES-1-resistance cells treated with CDDP. *P < 0.05.

Then we further investigated the effect of DLX6-AS1 on secondary cisplatin resistant of LUSC cells. CCK8 results showed that the IC50 of CDDP in SK-MES-1 cells was 0.63 μg/mL, and 7.72 μg/mL in SK-MES-1-resistance cells (Fig. 4a). Then we found that DLX6-AS1 expression was higher in SK-MES-1-resistance cells than that in SK-MES-1 cells (Fig. 4b), and was major in the cytoplasm of SK-MES-1-resistance cells (Fig. 4c). Further experiments showed that cisplatin treatment (7 μg/mL for resistant cells and 0.5 μg/mL for SK-MES-1 cells) significantly decreased DLX6-AS1 expression both in SK-MES-1 cells and SK-MES-1-resistance cells (Fig. 4d,e). According to UCSC prediction, H3K4me1 was enriched in the promoter region of DLX6-AS1 (Fig. 4f). Then Western blot analysis indicated that H3K4me1 was significantly up-regulated in SK-MES-1-resistance cells (Fig. 4g  and Supplementary Figure S1). Further ChIP assay confirmed that H3K4me1 was abundantly enriched in SK-MES-1-resistance cells (Fig. 4h). In addition, CDDP treatment markedly decreased the expression and enrichment of H3K4me1 in SK-MES-1-resistance cells (Fig. 4i,j  and Supplementary Figure S1). Therefore, the above results suggested that DLX6-AS1 expression was induced by H3K4me1 in cisplatin resistant LUSC cells.

### DLX6-AS1/miR-181a-5p/miR-382-5p regulation influenced the effect of secondary cisplatin resistance in LUSC cells

**Figure 5:**
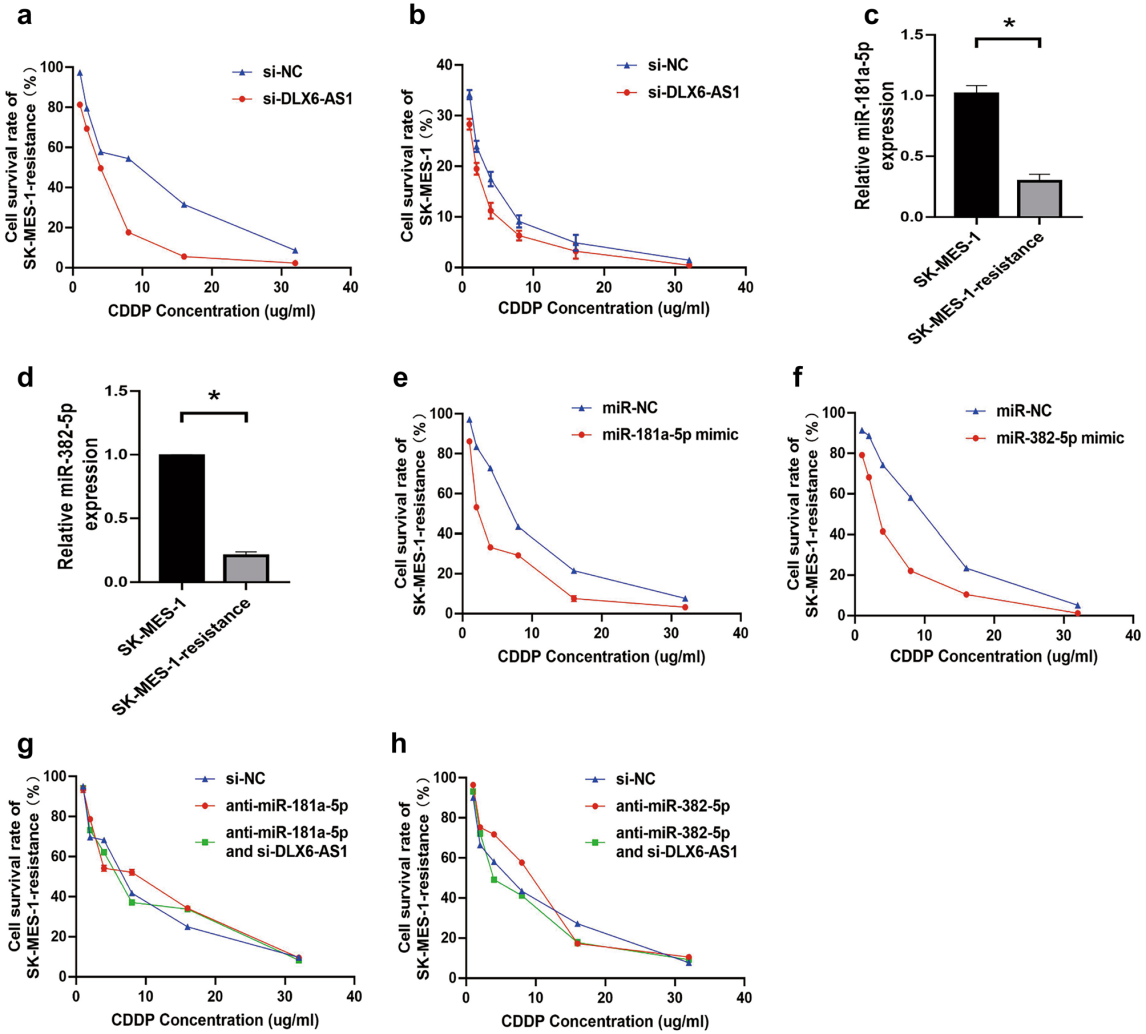
DLX6-AS1/miR-181a-5p/miR-382-5p regulation influenced the effect of secondary cisplatin resistance in LUSC cells. (**a**) CCK-8 assay for the IC50 of SK-MES-1-resistance cells transfected with si-DLX6-AS1 or si-NC and treated with CDDP. (**b**) CCK-8 assay for the IC50 of SK-MES-1 cells transfected with si-DLX6-AS1 or si-NC and treated with CDDP. (**c,d**) qRT-PCR analysis for the relative expression of miR-181a-5p (**c**) or miR-382-5p (**d**) in SK-MES-1 or SK-MES-1-resistance cell lines. (**e,f**) CCK-8 assay for the IC50 of SK-MES-1-resistance cells transfected with miR-181a-5p mimic (**e**), miR-382-5p mimic (**f**) or their respective miR-NC and treated with CDDP. (**g**) CCK-8 assay for the IC50 of SK-MES-1-resistance cells transfected with anti-miR-181a-5p or si-DLX6-AS1 and treated with CDDP. (**h**) CCK-8 assay for the IC50 of SK-MES-1-resistance cells transfected with anti-miR-382-5p or si-DLX6-AS1 and treated with CDDP. *P < 0.05.

After inhibiting the expression of DLX6-AS1, we noticed that the IC50 of SK-MES-1-resistance (IC50: si-NC, 7.18 ug/mL vs. si-DLX6-AS1, 3.17 ug/mL) and SK-MES-1 cells (IC50: si-NC, 0.60 ug/mL vs. si-DLX6-AS1, 0.43 ug/mL) was significantly decreased, indicating that DLX6-AS1 might regulate secondary cisplatin resistance in LUSC cells (Fig. 5a,b). Moreover, the expression of miR-181a-5p and miR-382-5p was significantly lower in SK-MES-1-resistance cells than that in the parental cells (Fig. 5c,d). Then we up-regulated the expression of miR-181a-5p and miR-382-5p in SK-MES-1-resistance cells, CCK8 results showed that the IC50 of SK-MES-1-resistance cells was remarkably decreased (IC50: miR-NC, 6.83 ug/mL vs. miR-181a-5p mimic, 3.59 ug/mL; miR-NC, 6.44 ug/mL vs. miR-382-5p mimic, 3.07 ug/mL; Fig. 5e,f). Further rescue experiments showed that inhibition of miR-181a-5p and miR-382-5p partially rescued the decreased IC50 caused by DLX6-AS1 knockdown in SK-MES-1-resistance cells (IC50: si-NC, 5.82 ug/mL vs. anti-miR-181a-5p, 8.58 ug/mL, anti-miR-181a-5p and si-DLX6-AS1, 5.81 ug/mL; si-NC, 5.38 ug/mL vs. anti-miR-382-5p, 9.29 ug/mL, anti-miR-382-5p and si-DLX6-AS1, 4.81 ug/mL; Fig. 5g,h). Taken together, these results indicated that DLX6-AS1/miR-181a-5p/miR-382-5p regulation influenced the effect of secondary cisplatin resistance in LUSC cells.

### CELF1 served as the target of miR-181a-5p and miR-382-5p

**Figure 6:**
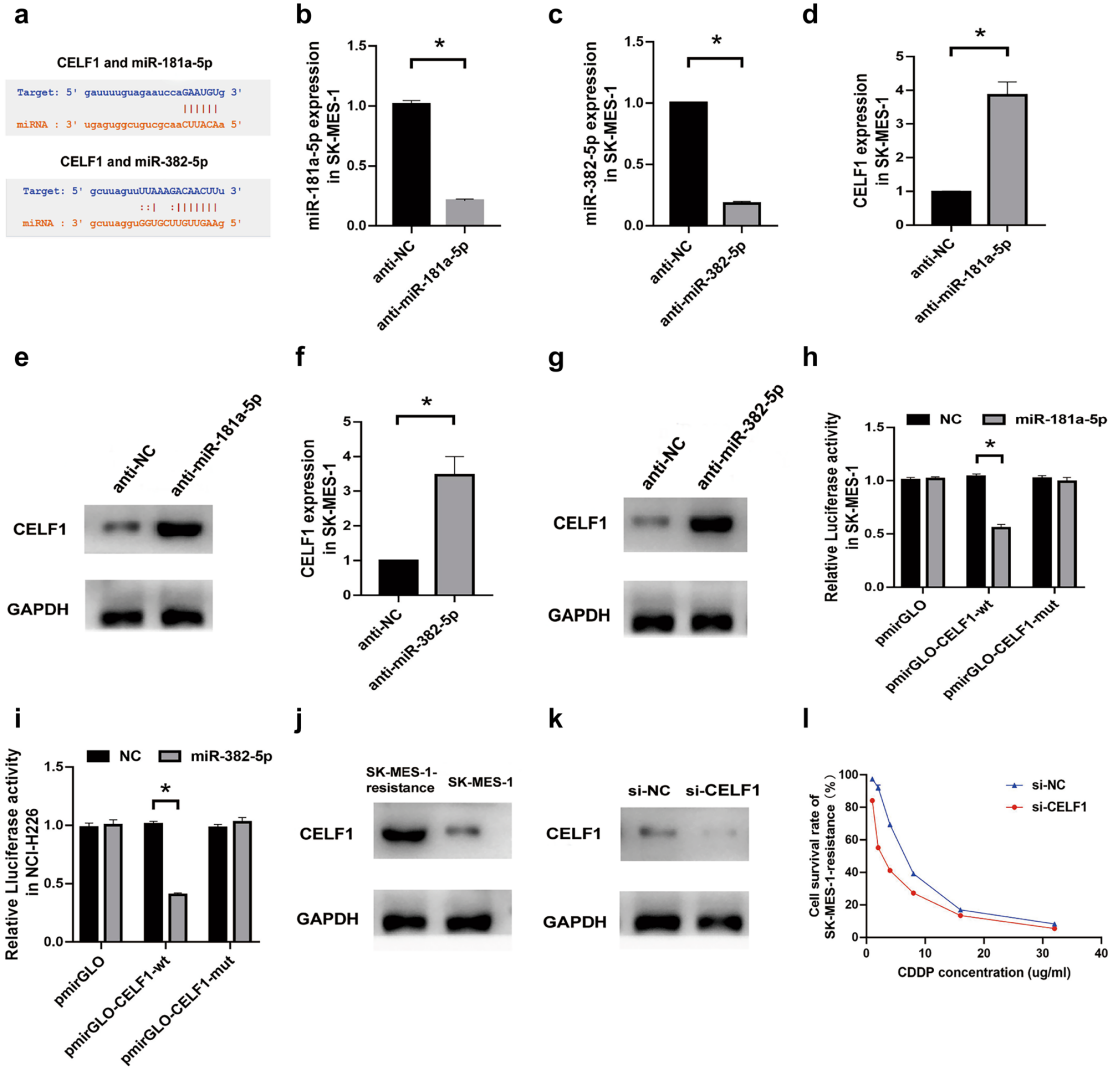
CELF1 served as the target of miR-181a-5p and miR-382-5p. (**a**) The binding sites of CELF1 with miR-181a-5p or miR-382-5p predicted by Starbase. (**b**) qRT-PCR analysis for the relative expression of miR-181a-5p in SK-MES-1 cells transfected with anti-miR-181a-5p or anti-NC. (**c**) qRT-PCR analysis for the relative expression of miR-382-5p in SK-MES-1 cells transfected with anti-miR-382-5p or anti-NC. (**d,e**) qRT-PCR, (**d**) analysis and Western blot, (**e**) analysis for the relative expression of CELF1 in SK-MES-1 cells transfected with anti-miR-181a-5p or anti-NC. (**f,g**) qRT-PCR, (**f**) analysis and Western blot, (**g**) analysis for the relative expression of CELF1 in SK-MES-1 cells transfected with anti-miR-382-5p or anti-NC. (**h**) The binding relationship between CELF1 and miR-181a-5p in SK-MES-1 cells was verified by performing Dual luciferase reporter gene assay. (**i**) The binding relationship between CELF1 and miR-382-5p in NCI-H226 cells was verified by performing Dual luciferase reporter gene assay. (**j**) The relative expression of CELF1 in SK-MES-1-resistance or SK-MES-1 cells was estimated by Western blot analysis. (**k**) The relative expression of CELF1 in SK-MES-1-resistance transfected with si-CELF1 or si-NC was estimated by Western blot analysis. (**l**) CCK-8 assay for the survival of SK-MES-1-resistance cells transfected with si-CELF1 or si-NC and treated with CDDP. *P < 0.05.

Based on Starbase, CELF1 was predicted as the potential target of miR-181a-5p and miR-382-5p (Fig. 6a). Then, we inhibited the expression of miR-181a-5p and miR-382-5p in SK-MES-1 cells and verified the inhibition efficiency by performing qRT-PCR analysis (Fig. 6b,c), and found that CELF1 expression was significantly increased both at mRNA and protein levels (Fig. 6d–g and Supplementary Figure S2). Furthermore, Dual luciferase reporter gene assay showed that miR-181a-5p and miR-382-5p directly interacted with CELF1 (Fig. 6h,i). In addition, CELF1 was found to be overexpressed in SK-MES-1-resistance cells (Fig. 6j and Supplementary Figure S2). Then we suppressed CELF1 expression in SK-MES-1-resistance cells (Fig. 6k and Supplementary Figure S2) and found that the IC50 was significantly decreased in SK-MES-1-resistance cells (IC50: si-NC, 6.70 ug/mL vs. si-CELF1, 2.96 ug/mL; Fig. 6l). Thus, these findings indicated that CELF1 was the target of miR-181a-5p and miR-382-5p in secondary cisplatin resistance LUSC cells.

## Discussion

Hitherto, the standard chemotherapy regimen for LUSC is still based on platinum-containing alkylating agents^30^. Although most LUSC patients initially respond well to chemotherapy, the clinical outcome of LUSC remains extremely poor due to inevitably recurrence and development of secondary chemoresistance^31^. In recent years, a large number of genome-wide studies verified that lncRNAs played a vital role in the chemoresistance of tumor cells^32^. For instance, lncRNA RAD51-AS1 reduced the activity of DNA damage repair pathway involved in RAD51 to increase the etoposide sensitivity of HCC cells^33^. LncRNA LINC00461 negatively regulated the expression of miR-411-5p to mediate docetaxel resistance in breast cancer^34^. In present research, we found that lncRNA DLX6-AS1 was highly expressed in LUSC and local recurrence tissues, and overexpressed in LUSC cell lines. Additionally, survival analysis indicated that a high level of DLX6-AS1 was closely correlated with worse cancer-specific survival. Thus, DLX6-AS1 was selected to deeply study its role in secondary chemoresistance of LUSC.

Recently, increasing studies demonstrated that lncRNAs sponged the specific miRNAs to regulate the initiation, progression, and therapeutic response of lung cancer^35–37^. For example, lncRNA MALAT1 sponged miR-374b-5p to regulate the proliferation, migration, and invasion of NSCLC cells by upregulating SRSF7^38^. LncRNA MEG3 was overexpressed in NSCLC tissues and positively regulated SOX7 expression to induce cisplatin sensitivity of NSCLC cells by targeting miR-21-5p^39^. Our study found that DLX6-AS1 had a higher expression in LUSC tissues and cell lines, while miR-181a-5p and miR-382-5p expression were at a lower level in LUSC tissues and cell lines. Furthermore, DLX6-AS1 knockdown restrained proliferation, invasion, migration, and promoted apoptosis of LUSC cells. Recent studies of DLX6-AS1 involved in cell growth and apoptosis of breast cancer and renal cell carcinoma uncovered similar results^40,41^. The further assay showed that miR-181a-5p and miR-382-5p were up-regulated after DLX6-AS1 inhibition. Accordingly, Dual luciferase reporter gene assay and RNA pull-down assay further demonstrated that DLX6-AS1 directly interacted with miR-181a-5p and miR-382-5p. Therefore, our results indicated that DLX6-AS1 negatively modulated the expression of miR-181a-5p and miR-382-5p.

Then we explored the potential effect of DLX6-AS1 in LUSC secondary chemoresistance and found that DLX6-AS1 expression was increased in CDDP resistance LUSC cells, while its expression was reversed after treated with CDDP. Interestingly, further experiments revealed that H3K4me1 was enriched at the DLX6-AS1 promoter in CDDP resistance LUSC cells, and this trend was partially reversed by CDDP treatment. This finding was important because the methylation and demethylation of histone precisely regulated specific regulatory processes by activating or inhibiting the expression of certain cancer-related genes^42^. For example, histone methylation around the PTEN promoter inhibited its protein expression in colon cancer and melanoma^43^. The JMJD2D histone demethylase was recruited at the p21 promoter, which led to up-regulated p21 expression in liver cancer^44^. The present results indicated that H3K4me1 was abundant at the promoter of DLX6-AS1 and was inhibited by CDDP treatment, suggesting that histone methylation modification played a crucial role in mediating the transcription of DLX6-AS1 in the CDDP resistance process of LUSC. Then we suppressed DLX6-AS1 expression in CDDP resistance LUSC cells and found that the CDDP resistance index was significantly decreased, indicating the promoting role of DLX6-AS1 in LUSC chemoresistance. Moreover, miR-181a-5p and miR-382-5p expression were lower in CDDP resistance LUSC cells, and their overexpression significantly enhanced the cisplatin sensitivity of CDDP resistance LUSC cells, whereas their knockdown partially reversed this trend. Previous studies highlighted the tumor suppressor function of miR-181a-5p and miR-382-5p in regulating the progression and chemoresistance of several malignant tumors. Yang et al. revealed that miR-181a-5p enhanced the sensitivity of esophageal adenocarcinoma cells to cisplatin^45^. Zheng et al. suggested that miR-382-5p inhibited proliferation and invasion of breast cancer through regulating SNHG1 expression^46^. Similarly, our research also illustrated that miR-181a-5p and miR-382-5p were down-regulated in LUSC tissues and cell lines, and miR-181a-5p and miR-382-5p overexpression remarkably restrained the effect of platinum on cell viability of LUSC, which was reversed by the depletion of miR-181a-5p and miR-382-5p.

Generally, miRNAs could bind to the 3′ UTR region of a specific mRNA to restrain its transcription, thereby affecting its function^47^. In present study, CELF1 was predicted as the potential target of miR-181a-5p and miR-382-5p, and the interaction relationship was verified by qRT-PCR, Western blot, and Dual luciferase reporter gene assay. Former investigations revealed that CELF1 could enhance cell migration, invasion, and chemoresistance in colorectal cancer by targeting ETS2^28^. CELF1 was abundantly expressed in the glioma tissues, and CELF1 expression was repressed by miR-330-3p to inhibit proliferation and migration of glioma cells^27^. Similarly, our results also indicated that CELF1 was overexpressed in CDDP resistance LUSC cells, and CELF1 attenuation led to the decline of CDDP resistance in LUSC cells.

In conclusion, this research was the first to illustrate that DLX6-AS1 was highly expressed in LUSC tissues and cell lines, and the aberrant expression of DLX6-AS1 was tightly associated with proliferation, apoptosis, and local recurrence of LUSC. Moreover, our results demonstrated that DLX6-AS1 induced by H3K4me1 facilitated platinum-based chemoresistance in LUSC through interacting with miR-181a-5p/miR-382-5p/CELF1 axis. Hence, these findings identified the pivotal role of DLX6-AS1 in the progression and secondary chemoresistance in LUSC and provided a novel therapeutic target for patients with LUSC.

## Methods

### Study subjects

153 LUSC patients diagnosed based on pathology results were recruited from 2011 to 2013 in the First Affiliated Hospital of Xi’an Jiaotong University. Every recruited patient was informed about the pros and cons of acquiring their tissues for academic research. And all of the patients had signed the written informed consents. Our study was approved by the Ethics Committee of the First Affiliated Hospital of Xi’an Jiaotong University (Approval number: H-582). And all experiments were conducted in accordance with relevant guidelines and regulations, which consistent with the Declaration of Helsinki regulations.

### Cell culture

SK-MES-1, NCI-H226, and MRC-5 cell lines were purchased from American type culture collection (ATCC, USA). DMEM medium (HyClone, USA) supplemented with 10% fetal bovine serum (Gibco, Rockville, MD) and 1% penicillin–streptomycin (HyClone, USA) in a 5% CO_2_ incubator at 37 °C. The SK-MES-1 cells were then treated with continuous low-dose of cisplatin in a stepwise manner to developed cisplatin resistant SK-MES-1 (SK-MES-1-resistance) cells. This process was repeated until cells could stably survive in 1 µg/mL cisplatin medium. Then we increased the concentration of cisplatin to 2, 4, 6, 8 and 10 µg/mL. Resistance Index (IC50 of resistant cells/IC50 of parental cells) was used to evaluate the resistance ability. Resistance Index larger than 5 was considered resistant.

### Cell transfection

The small interference RNAs against DLX6-AS1 (si-DLX6-AS1), CELF1 (si-CELF1), and their corresponding negative control (si-NC) were synthesized by Shanghai Genechem Chemical Technology Co., Ltd (Shanghai, China). The miR-181a-5p mimic, miR-382-5p mimic, miR-181a-5p inhibitor (anti-miR-181a-5p), and miR-382-5p inhibitor (anti-miR-382-5p), as well as their respective negative control (miR-NC and anti-miR-NC) were acquired from GenePharma (Shanghai, China). Then the siRNAs (50 nM per well in a 24-well) above were transfected into SK-MES-1 and NCI-H226 cell lines by using the Lipofectamine 3000 (Invitrogen, USA) reagent according to the manufacturer’s instruction.

### qRT-PCR

RNeasy Mini Kit (Qiagen, Leusden, Netherlands) was used to extract the total RNA extraction. PrimeScript™ RT reagent Kit (Takara Biotechnology Ltd., China) was used to reverse transcribe the extracted the RNA into cDNA. As for miRNAs, One Step PrimeScript™ miRNA cDNA Synthesis Kit was used for reverse-transcription. SYBR Premix Ex Taq II kit (TaKaRa, Shiga, Japan) was used for the qRT-PCR process. 2−ΔΔCt method was used to quantify the results. U6 and GAPDH were used as the internal control for miRNAs and genes respectively. The primers are as follows: DLX6-AS1 F: 5′-AGTTTCTCTCTAGATTGCCTT-3′ R: 5′-ATTGACATGTTAGTG CCCTT-3′; miR-181a-5p: F: 5′-GGGCAGCCTTAAGAGGA-3′ R: 5′-CAGTGCGTGTCGTGGA-3′; miR-382-5p: F:5′-ATCCGTGAAGTTGTTCGTGG-3′ R: 5′-TATGGTTGTAGAGGACTCCTTGAC-3′; GAPDH: F: 5′-CACTGGGCTACACTGAGCAC-3′ R: 5′-AGTGGTCGTTGAGGGCAAT-3′; U6: F: 5′-CTCGCTTCGGCAGCACA-3′ R: 5′-AACGCTTCACGAATTTGCGT-3′.

### Isolation of cytoplasmic and nuclear RNA

Cytoplasmic & Nuclear RNA Purification Kit (Norgen, Canada) was used to isolate cytoplasmic and nuclear RNA. According to the manufacturer’s guidance, lysis buffer J was used to lyse the target cells. Then the cell lysis was centrifuged; the supernatant was used to extract the cytoplasmic fraction and a pellet was used to extract the nuclear fraction. Buffer SK and ethanol were used to purify and extract the cytoplasmic and nuclear RNA.

### Western blot analysis

The total proteins were extracted by using radioimmunoprecipitation (RIPA) lysis buffer added with phosphatase inhibitors (Roche, Switzerland) and protease inhibitors (Roche, Switzerland). After being quantified by using a bicinchoninic acid assay (BCA) protein assay kit (Beyotime, Shanghai, China), the proteins were separated by sodium dodecyl sulfate polyacrylamide gel electrophoresis (SDS-PAGE), and then transferred to a polyvinylidene fluoride (PVDF) membrane (Vazyme). 5% skimmed milk was used for transferred PVDF membranes block for 1 h at room temperature. And then the membranes were incubated overnight at 4 °C with the primary antibodies, including H3K4me1 (1:1000, Cell Signaling Technology, USA), Histone (1:2000, Cell Signaling Technology, USA), CELF1 (1:2000, Proteintech, USA), and GAPDH (1:5000, Proteintech, USA). The membranes were then incubated with the secondary antibody (1:10,000, Beyotime, Shanghai, China) labeled with horseradish peroxidase at room temperature for 2 h. Then the enhanced chemiluminescence (ECL, Beyotime, Shanghai, China) was used to evaluate the protein expression levels.

### Transwell assay

To investigate cell invasion and migration abilities, cells were resuspended in a serum-free medium and plated onto the upper chamber of a Matrigel-coated Transwell insert (EMD Millipore). Complete medium with 10% FBS was added to the lower chamber. The plate was incubated in a CO_2_ incubator at 37 °C. After 24 h, the upper layer of the membrane was wiped with a cotton swab, and cells attached to the lower surface were fixed with 4% paraformaldehyde for 20 min, and stained with 1% 4′, 6-diamidino-2-phenylin-dole (DAPI) solution for 15 min. Then cells were observed and counted under an inverted microscope.

### Cell apoptosis assay

Annexin V-FITC/PI double Kit (BD Bioscience, USA) was used to test the apoptosis rate of SK-MES-1 cell line. SK-MES-1 cells were seeded on the 6-well plates and washed with cold PBS after 24 h, and then suspended in 100 μL binding buffer. The cells were subsequently stained with 5 μL annexin V-FITC and 5 μL propidium iodide (PI) and incubated for 15 min at room temperature in dark. Finally, the apoptosis rate of SK-MES-1 cells was analyzed by flow cytometry (ThermoFisher Scientific, USA).

### Cell counting kit-8 (CCK-8) assay

Cell viability was investigated by performing CCK-8 (APExBIO, USA) assay according to the manufacturer’s protocol. LUSC cells were resuspended and seeded in a 96-well plate at the concentration of around 1 × 10^3^/well. Cells were then cultured at the 5% CO_2_ and 37 °C with DMEM medium for 24 h, 48 h, 72 h, 96 h, and 120 h respectively. Each well was added with 10 μL CCK-8 reaction solution followed by incubation for 2 h. Finally, the optical density (OD) values at 450 nm were measured.

### RNA pull-down assay

Biotinylated probes for miR-181a-5p-WT, miR-181a-5p-Mut, miR-382-5p-WT, and miR-382-5p-Mut were constructed by Genecreate. The miR-181a-5p-Mut and miR-382-5p-Mut refer to the mutations in the binding sites between DLX6-AS1 and miR-181a-5p and miR-382-5p. M-280 Streptavidin-coated MagneSphere particles were used to harvest the bonded RNA, which was then eluted, harvested and purified. Finally, qRT-PCR was used to investigate the DLX6-AS1 expression level.

### Dual luciferase reporter gene assay

The binding site sequences of DLX6-AS1, miR-181a-5p, and miR-382-5p were cloned into a luciferase reporter vector (Promega, Madison, WI, USA). Then the plasmids of pmirGLO-DLX6-AS1 wild type (WT) or pmirGLO-DLX6-AS1 mutant type (Mut) were co-transfected with the miR-181a-5p/NC and miR-382-5p/NC mimic into SK-MES-1 and NCI-H226 cells by using Lipofectamine 2000 (Invitrogen, USA) for 48 h. Then the luciferase activities were estimated by applying a Dual luciferase reporter analysis system (Promega, USA) based on the manufacturer’s instructions.

### Chromatin immunoprecipitation (ChIP) assay

ChIP assay was conducted by using an EpiQuik ChIP kit (Epigentek, USA) according to the instruction of the manufacturer. Briefly, LUSC cells were fixed with 1% formaldehyde at room temperature for 10 min. Then cells were added with 1/10 volume of 1.25 M glycine to stop fixation and incubated for 5 min. Then added 5 mL cell lysis buffer to resuspend the cell pellet and lysed on ice for 10 min. After centrifugation, cells were added with 1 ml ChIP buffer (added 12 μL PMSF and 10 μL protease inhibitor for every 1 mL ChIP buffer) for sonication (10 min, 10 s on, 10 s off). Then 200 µg of the protein-chromatin complex was extracted for each immunoprecipitation, and the pre-blocked dynabeads protein G (Invitrogen) was used to capture antibody-protein complexes. The eluted product was purified with a DNA purification kit (Invitrogen) to obtain ChIP DNA. Then the ChIP DNA was analyzed by qPCR. The antibodies included: H3K4me1 (Cell Signaling Technology, USA), and normal mouse IgG (Epigentek, USA).

### Statistics analysis

All data involved in this study were processed and visualized by using the SPSS 18.0 software and the Graphpad Prism 8.2 software. The results presented were expressed as Mean ± Standard Deviation (SD). A paired Student’s t-test was used for comparison between two groups. One-way ANOVA was conducted to compare the differences among multiple groups. Survival analysis was performed by applied Kaplan–Meier analysis. Each experiment in this research was independently repeated at least three times. A *P* value of less than 0.05 was considered to indicate a statistically significant difference.

## Supplementary Information

Supplementary Information.
