# Supplementary material for: DLX6-AS1 activated by H3K4me1 enhanced secondary cisplatin resistance of lung squamous cell carcinoma through modulating miR-181a-5p/miR-382-5p/CELF1 axis
Source: Sci Rep. 2021 Oct 25;11:21014. doi: 10.1038/s41598-021-99555-8 (PMC8546124; doi:10.1038/s41598-021-99555-8)
Supplement: Supplementary file 1 — Supplementary Information. [file 41598_2021_99555_MOESM1_ESM.pdf]

Title： DLX6-AS1 activated by H3K4me1 enhanced secondary cisplatin resistance of lung squamous cell carcinoma through modulating miR-181a-5p/miR-382-5p/CELF1 axis

Author details：

Xu Zhao, Department of Radiation Oncology, The First Affiliated Hospital of Xi'an Jiaotong University, Xi'an, Shaanxi, 710061, China. e-mail: zx19890305@stu.xjtu.edu.cn;

Jizhao Wang, Department of Thoracic Surgery, The First Affiliated Hospital of Xi'an Jiaotong University, Xi'an, Shaanxi, 710061, China. e-mail: wangjz1104@xjtu.edu.cn;

Rui Zhu, Department of Thoracic Surgery, The First Affiliated Hospital of Xi'an Jiaotong University, Xi'an, Shaanxi, 710061, China. e-mail: 1261093968@qq.com;

Jing Zhang, Department of Thoracic Surgery, The First Affiliated Hospital of Xi'an Jiaotong University, Xi'an, Shaanxi, 710061, China. e-mail: 008147@xjtufh.edu.cn;

Corresponding Author: Yunfeng Zhang, Department of Thoracic Surgery, The First Affiliated Hospital of Xi'an Jiaotong University, No.277, Yanta West Road, 710061, Xi'an, Shaanxi, China. e-mail: zyf100@xjtu.edu.cn

Supplementary Figure S1.

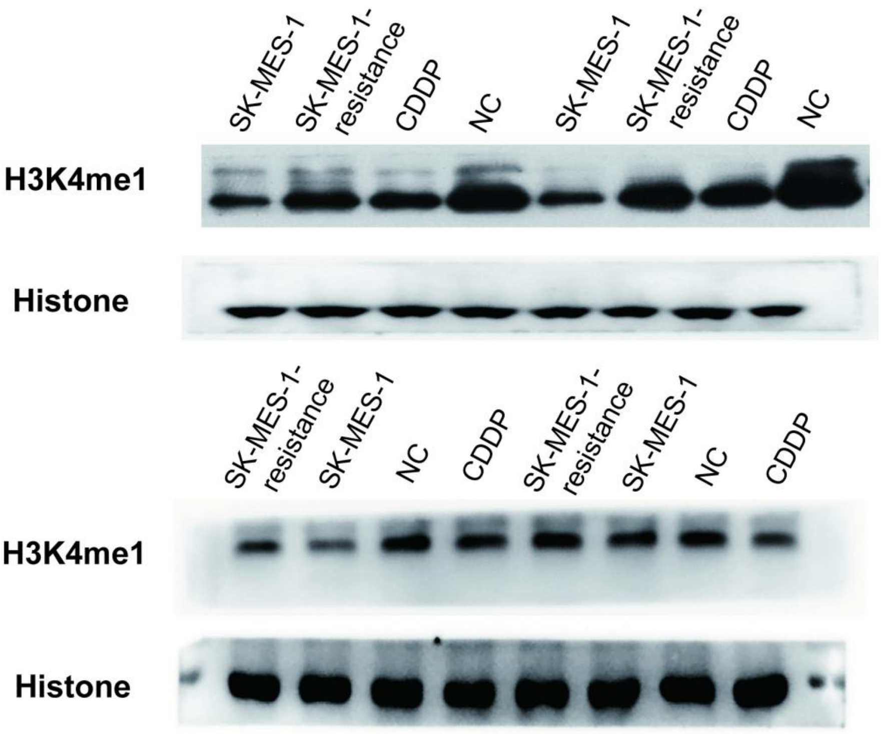

Supplementary Figure S1. The scans of membranes for Western blot images of H3K4me1 expression in SK-MES-1 cells, SK-MES-1-resistance cells, or SK-MES-1-resistance cells treated with or without CDDP respectively corresponding to Fig. 4g and Fig. 4i.

Supplementary Figure S2.

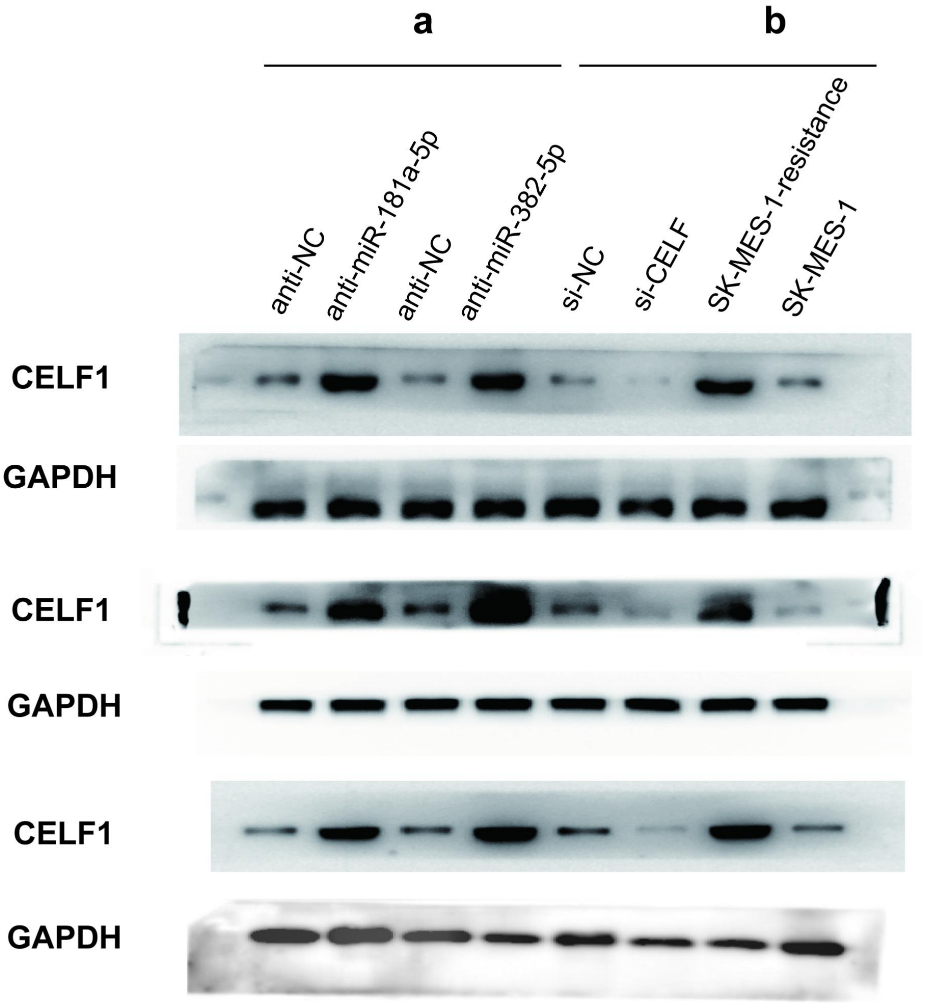

Supplementary Figure S2. The scans of membranes for Western blot images of CELF1 expression. (**a**) The relative expression of CELF1 in SK-MES-1 cells transfected with anti-miR-181a-5p, anti-miR-382-5p or their respective anti-NC corresponding to Fig. 6e and Fig. 6g. (**b**) The relative expression of CELF1 in SK-MES-1 cells, SK-MES-1-resistance cells, or SK-MES-1-resistance cells treated with si-CELF1 or si-NC respectively corresponding to Fig. 6j and Fig. 6k.
